# Serological Investigation of* Peste Des Petits Ruminants* in East Shewa and Arsi Zones, Oromia Region, Ethiopia

**DOI:** 10.1155/2017/9769071

**Published:** 2017-12-14

**Authors:** Getachew Gari, Biressaw Serda, Dejene Negesa, Fethu Lemma, Hagos Asgedom

**Affiliations:** ^1^National Animal Health Diagnosis and Investigation Center, Sebeta, Ethiopia; ^2^College of Veterinary Medicine, Haramaya University, P.O. Box 138, Dire Dawa, Ethiopia

## Abstract

*Peste des petits ruminant (PPR)* is an economically important disease of small ruminants with a rapidly expanding geographical distribution. There are fragmented reports to the occurrence and distribution of the disease in Ethiopia. A total of 700 serum samples were collected from goats and sheep to detect the presence of antibody against PPR virus using Competitive Enzyme-Linked Immunosorbent Assay (C-ELISA). An overall PPR seropositivity was reported to be 48.43% in the area. There is no statistically significant difference in the seroprevalence of the disease between sheep and goats (50.85% and 46.68%), respectively. However, there was statistically significant variation (*P* < 0.05) in the seroprevalence of the disease in young (33.9%) and adult (55.8%) age categories. The seroprevalence in male and female was 42.07% and 50.09%, respectively, where the variation was statistically not significant (*P* > 0.05). High seroprevalence of* Peste des petites ruminants* in the study area indicated the virus circulation and endemicity of the disease. The disease causes substantial economic losses by affecting the livelihood of the farmers. Therefore, control measures should be put in place to minimize the loss associated with the disease.

## 1. Introduction

*Peste des petits ruminant (PPR)* is an acute, highly contagious, and frequently fatal viral disease of sheep, goats, and wild small ruminants. It is characterized by fever, mucopurulent ocular and nasal discharges, necrotizing and erosive stomatitis, severe enteritis, and pneumonia leading to death [1, 2]. PPR is a transboundary animal disease of significant economic importance, ranking among the top ten diseases affecting small ruminants [3, 4]. Sheep and goat populations are estimated to be 25.5 million and 26.43 million, respectively, in Ethiopia and this is the largest population in Africa [5].

The causal agent, PPR virus (PPRV), is an enveloped ribonucleic acid virus of the genus* Morbillivirus* of family Paramyxoviridae [6]. The disease was first reported in Cote d'Ivoire in 1942 [2] and it has spread across the Sub-Saharan Africa, Morocco, Arabian Peninsula, Middle East, Turkey, Iran, Iraq, Pakistan, India, Bangladesh, Nepal, Tajikistan and Kazakhstan, Tibet, and China [7–10].

PPR was clinically suspected for the first time in Ethiopia in 1977 in a goat herd from Afar region, eastern part of the country [11]. Clinical and serological evidence of its presence has been reported by Taylor (1984) [12] and later confirmed in 1991 with cDNA probe in lymph nodes and spleen specimens collected from an outbreak in a holding near Addis Ababa [13]. Abraham et al. (2005) reported the overall seroprevalence of 9% in goats and 13% in sheep in different parts of Ethiopia [14]. It was also reported that 14.6% of sheep sampled along 4 roads from Debre Berhan to Addis Ababa were seropositive [15]. In 1999 national serosurveillance of PPR conducted in Ethiopia, the overall seroprevalence of 6.4% (95% CI : 6.0–6.8) in both goats and sheep ranging from 0% to 52.5% was estimated [15].

In 1997 one study revealed up to 100% of seropositive individuals in groups of adult male sheep and animals that survived suspected outbreaks [13]. Based on the reported morbidity and mortality of the infection and the size and structure of the small ruminant sector, it is likely that PPR became one of the most economically important livestock diseases in the country [16].

Studies so far conducted provide historical information about the frequency and distribution of PPR in Ethiopia and yet suggest extensive circulation of PPR virus among the small ruminant population. Therefore, the objective of current study was to estimate the seroprevalence of* peste des petits ruminants* in sheep and goats using serological tests.

## 2. Materials and Method

### 2.1. Study Area

**Figure 1:**
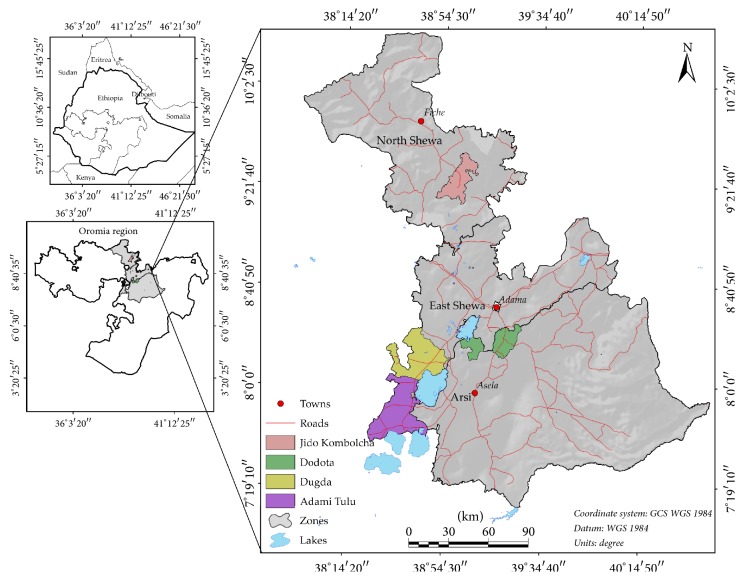
Map of study area.

The study was conducted in eight Kebeles (the lowest administrative level in Ethiopia), distributed in three districts (Dugda and Adami Tullu Districts) in Eastern Showa Administrative Zone and Dodota District in Arsi Zone of Oromia regional state, Ethiopia. Arsi is one of the zones of Oromia Region and 186 km from Addis Ababa. It has a latitude of 7°45′0^″^N and longitude of 39°30′0^″^E. Arsi has annual rain fall range from 700 to 950 mm. Dodota is part of Arsi Zone district. Dodota is located in Great Rift Valley. The altitude of the district ranges from 140 to 250 m above sea level. On the other hand, Dugda and Adami Tullu are Districts in the eastern Showa Zone of Oromia Regional state. Adami Tullu has latitude of 7°52′0^″^ North and longitude of 38°42′0^″^ East with elevation of 1643 meters above sea level. Dugda has a latitude of 8°47′0^″^ North and longitude of 38°18′0^″^ East with elevation of 1636 meters above sea level (Figure 1).

### 2.2. Study Design and Animal Population

Cross-sectional study was conducted from November 2014 to March 2015 to determine the seroprevalence of PPR in small ruminants. The animals were reared in a mixed crop-livestock farming system but have not been vaccinated before sample collection. Epidemiological data related to risk factors associated with PPR occurrence such as sex and age of sheep and goats were collected by using a checklist. All age groups of goats and sheep were sampled but young age greater than six months was considered for sampling to rule out maternal antibody. In this study, young age groups are animals from 6 months to 18 months and adult age is defined as animals more than 18 months per example. There was no history/record of vaccination against PPR in the study so far.

### 2.3. Sampling Strategy and Sample Collection

The study involved multistage random sampling where districts laying in the Great Rift Valley segment of Ethiopia were selected purposely for their high small ruminant population, but each kebele was first randomly selected and animal unit was selected randomly at last. The blood samples were collected from the jugular vein using sterile vacutainer tubes and kept at room temperature to clot down for 12 hrs. The serum was extracted using centrifuge spun at 2000 rpm and stored in ice packs +4°C until transported to the National Animal Health Diagnosis and Investigation Center, Sebeta, Ethiopia. The samples were stored at −20°C deep freeze in the laboratory until the test is conducted.

### 2.4. Sample Size Determination

The sample size was determined using Thrusfield [17] formula. Since there was no prior similar study conducted in the area, expected seroprevalence of 50% was assumed to get the maximum number of samples sizes required. The absolute precisions were decided to be 5% and 95% confidence level. Thus, sample size estimation formula is shown below:

(1) $$\begin{array}{c} n=\frac{1.96^{2}P_{\exp}\left(1-P_{\exp}\right)}{d^{2}}, \end{array}$$

  where *n* is the required sample size,
 
*P*_exp_ is the expected seroprevalence,
 
*d*^2^ is the desired absolute precision.

Accordingly, a sample size of 384 was estimated using the formula. However, the sample size was increased to 700 samples sizes to increase the statistical efficiency and to consider the clustering effect in multistage sampling design. Out of 700 samples, 407 samples were collected from goats and 293 from sheep.

### 2.5. Serological Test for PPRV Specific Antibody

Competitive Enzyme-Linked Immunosorbent Assay (C-ELISA) was run to test the serum samples as prescribed by the manufacturer and the Office International des Epizooties Terrestrial Manual [18]. The laboratory test was conducted in National Animal Health Diagnostic and Investigation Centre (NAHDIC). The c-ELISA kit comprised PPR antigen (75/I) strain, anti-PPRV monoclonal antibody, anti-mouse conjugate, control sera, substrate, and chromogen. The ELISA test result was read at 492 nm wave length and the percentage inhibition (PI) value was calculated [19].

### 2.6. Data Management

The data collected were entered into Epi-Data version 3.0 and analyzed using SPSS version 16.0. For statistical significance, 95% CI, and *P* value of 0.05 and for existence of difference among different categories, chi-square values were considered.

## 3. Results

The overall seroprevalence of PPR in sheep and goats was 48.43% (95% CI: 32.27–56.32). The seroprevalence across the geographical location in each district was 54.88%, 45.90%, and 47.54% in Dodota, Adami Tullu, and Dugda, respectively (Table 1).

The association of age and sex groups to PPR seroprevalence occurrence showed that age factor was found significantly associated risk factor (*P* < 0.05) (Table 2).

## 4. Discussion

Serological result of this study showed an overall seroprevalence of 48.43% in two species in selected area of Eastern Showa and Arsi Zones of Oromia Regional state, which was close to the reports made from Nigeria with seroprevalence of 55% [20], from Uganda 55.2% [21], from Maychew district of Tigray regional state, Ethiopia, 43.6% [22]. The comparable seroprevalence 50% in Adigudam and Chercher of Tigray, Ethiopia, was reported [22]. The seroprevalence across sex variation in Tigray showed that 47.5% and 43.7% in female and male, respectively [22], were comparable to our finding with 50.09% in female and 42.07% in male. However, the seroprevalence in this study was lower than 67% in goat at Afar [23] and 61.8% in Sudan [24] maybe due to difference in agroecology. The result observed in the current study was higher than the report of 14.6% from Debra Berhan to Addis Ababa road, Ethiopia [25]. The difference in agroclimatic conditions, cultural and social practice, and different production systems could be the reason for the variations between the current report and the previous reports.

In the current study, seroprevalence of the disease among the study districts was observed. There was no statistical significant variation in seroprevalence between the study areas with 54.88%, 47.54%, and 45.90% in Dodota, Dugda, and Adami Tullu, respectively. The seroprevalence of PPR between sex groups showed that it was 50.09% in female and 42.07% in male but there was no statistically significant variation between sex groups. This is in agreement with previous finding [19].

In Ethiopia goats are affected more severely to PPR virus exposure compared to sheep and they exhibit striking clinical sign while sheep undergo mild form of the disease [26]. The seroprevalence in sheep (46.68%) was approximately the same with that of goats (50.85%) which may resulted from equal exposure of sheep and goat because they are herded together and communal grazing. Similar observation was reported with seroprevalence of 9% in goats and 13% in sheep [14] in pastoral production system of Ethiopia due to equal exposure.

In previous studies of PPRV isolates originating from Punjab Pakistan, a higher affinity of the virus for the ovine species over the caprine species has been observed [11]. An outbreak with a high mortality in sheep was also reported that sheep possessed an innate resistance to the clinical effects of disease, but occasional field strains could overcome this resistance and produce high mortality [26].

In conclusion, high seroprevalence of PPR in the study area and the high level of seroprevalence in adult small ruminants indicated the occurrence of virus circulation and the endemicity of the disease. The disease causes substantial economic losses by affecting the livelihood of the farmers. Therefore, control measures should be put in place to minimize the loss associated with the disease.

## Figures and Tables

**Table 1:** Seroprevalence of PPR in sheep and goats from study area.

Factors	Number tested	Number positive (%)	*X* ^2^	*P* value
District				
Dodota	164	90 (54.88)	3.70	0.16
Adami Tullu	353	162 (45.90)		
Dugda	183	87 (47.54)		
Species				
Sheep	293	149 (50.85%)	1.19	0.276
Goat	407	190 (46.68%)		
Age				
Young	236	80 (33.90%)	30.10	0.000
Adult	464	259 (55.82%)		
Sex				
Female	555	278 (50.09%)	2.96	0.09
Male	145	61 (42.07%)		

*Total*	700	339 (48.43)		

**Table 2:** Seroprevalence of PPR occurrence in sheep and goats for different sex and age groups.

Species	Number tested	Number positive (%)	*𝒳* ^2^	*P* value
Sheep				
Age				
Young	84	32 (38.1)	7.67	0.006
Adult	209	117 (56.0)		
Sex				
Female	235	121 (51.5)	0.19	0.66
Male	58	28 (48.3)		
Goats				
Age				
Young	152	48 (31.6)	22.24	0.000
Adult	255	190 (74.5)		
Sex				
Female	320	157 (49.0)	3.41	0.07
Male	87	33 (37.9)		

*Total*	700	339 (48.43)		
